# Cohort Profile: The NSPN 2400 Cohort: a developmental sample supporting the Wellcome Trust NeuroScience in Psychiatry Network

**DOI:** 10.1093/ije/dyx117

**Published:** 2017-11-21

**Authors:** Beatrix Kiddle, Becky Inkster, Gita Prabhu, Michael Moutoussis, Kirstie J Whitaker, Edward T Bullmore, Raymond J Dolan, Peter Fonagy, Ian M Goodyer, Peter B Jones

**Affiliations:** 1 Department of Psychiatry, University of Cambridge, United Kingdom; 2 Wellcome Trust Centre for Neuroimaging, University College London, United Kingdom; 3 Cambridgeshire and Peterborough National Health Service Foundation Trust, Cambridge, United Kingdom; 4 Research Department of Clinical, Educational and Health Psychology, University College London, United Kingdom; 5 Max Planck University College London Centre for Computational Psychiatry and Ageing Research, University College London, United Kingdom; 6 Medical Research Council/Wellcome Trust Behavioural and Clinical Neuroscience Institute, University of Cambridge, United Kingdom; 7 ImmunoPsychiatry, GlaxoSmithKline Research and Development, Stevenage, United Kingdom

## Why was the cohort set up?

Mental and substance use disorders are the leading cause of years lived with disability, worldwide.^1^ Other than childhood developmental disorders and neurodegenerative dementias of the elderly, most mental health disorders are first manifest in the second and third decades of life during which the highest proportion of total disability adjusted life years occurs due to their enormous impact on normal, adolescent and young adult functioning;^1^ non-syndromal abnormalities can be identified far earlier in life.

The normal human brain undergoes a range of normative developmental process during this extended post-pubertal epoch, but the events that account for the massive increases in risk for mental health disorders remain obscure, something compounded by the questionable validity of current psychiatric nosology. Thus, the development of preventative or disease-modifying approaches remain a distant goal.

Recent applied neuroscience advances highlight three pathways of exploration in order to reconstruct nosology^2^: 1) studying the extent of variation in cognition and behaviour throughout the general population rather than comparing categories of mentally well and mentally ill; 2) investigating brain systems underlying emotion, cognition and behaviour; if these emerge from integration of activity over large-scale brain networks, it should be possible to mechanistically link the variation in psychological phenotypes with differences in underlying brain systems; 3) adopting a developmental perspective to understand optimal/suboptimal trajectories of neurocognition as early as possible within the high risk period.

We aimed to link normal and psychopathological variation at the behavioural, cognitive and emotion level to phenotypic variation at the level of brain systems, subverting the traditional division between adult and child/adolescent psychiatry by measuring specified dimensions in healthy volunteers and patients in the age range of 14–24 years.

The NSPN 2400 Cohort was established in July 2012 as a collaboration between the University of Cambridge and University College London supported primarily by a strategic award from the Wellcome Trust.

## Who is in the cohort?

The NSPN 2400 Cohort is a general population sample aged 14-24 years conceived to support an accelerated longitudinal design to measure developmental change. This design involves recruitment of multiple, age-adjacent cohorts followed longitudinally for a limited period of time, which permits estimation of trajectory across a wider range of ages more quickly than a single-cohort longitudinal follow-up.^3^ In addition to its efficiency, bias from attrition can be less problematic given that drop outs in cohorts is related to study duration, highlighting another advantage of the accelerated design.^4^

The NSPN 2400 Cohort aimed to recruit at least 2000 participants in an age- sex-stratified sample, including equal numbers of males and females for the following five age groups: 14-15, 16-17, 18-19, 20-21, and 22-24.99 years. Participants received a Home Questionnaire Pack (HQP) and Sociodemographic Questionnaire that focused on assessing participants’ mood, behaviour and wellbeing along with demographic characteristics. This was accompanied by an Oragene saliva sampling kit for DNA collection that was returned to the study team by post, together with the completed questionnaires.

**Figure 1:**
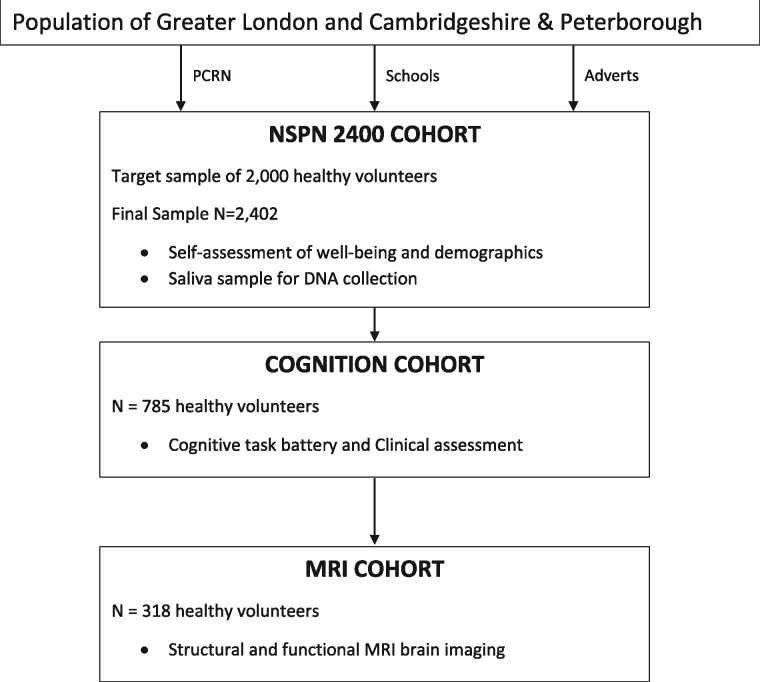
Predicted cascade sampling of study cohorts within the NSPN.

Two samples with more intensive measures are embedded within the NSPN 2400 Cohort (Figure 1). First, the ‘MRI cohort’ (N = 318) took part in in-unit assessments of brain structure and function, using magnetic resonance imaging (MRI), as well as detailed behavioural assessments of cognitive and social cognitive function using computer-based evaluations, clinical assessments and IQ measures. Participants from each age- sex-stratum were invited in equal numbers using the order in which they had been recruited to the 2400 cohort (assumed to be random) until at least 30 from each stratum had been through the assessment. An additional sub-sample (N = 467) participated in the same computational tests of cognitive function and clinical assessments but without the MRI component. Again, these were recruited from the ten age-sex strata as for the MRI cohort, aiming for a sample size of at least 450 additional subjects with detailed cognition measurement and, including the MRI cohort, a total of 750 or more people with the cognitive assessments, This combined sub-sample with cognition measures (the ‘cognition cohort’) comprises 785 people, of which 318 (the MRI cohort) have both MRI and cognition measurements. When resources for taking blood allowed, participants in both cohorts were asked to provide a venous blood sample for future genetic, epigenetic and gene expression. The MRI and cognition cohorts were followed-up on one or two occasions. By the virtue of this design, there are participants that completed all three waves of HQP as well as three in-unit assessments.

## Recruitment

The NIHR Primary Care Research Network (PCRN) engaged 50 GP’s to recruit young people using their sex-age registers by sending out invitations (including an expressions of interest form (EoI)) across Cambridgeshire and Greater London (closest proximity to universities leading the study). Schools and Further Education colleges were also engaged to distribute the EoI forms to 14 to 18-year-old participants. The NSPN recruitment team assisted GP’s and schools by providing invitation to participate letters, which were forwarded to potential participant’s home address that remained unknown to the NSPN investigators. Purposive advertisement was also used during recruitment; invitation letters with EoI were sent to those who responded to advertisements that met the age criteria. If an individual wanted to participate they informed NSPN recruitment team over the phone/sent in completed EoI form.

**Figure 2:**
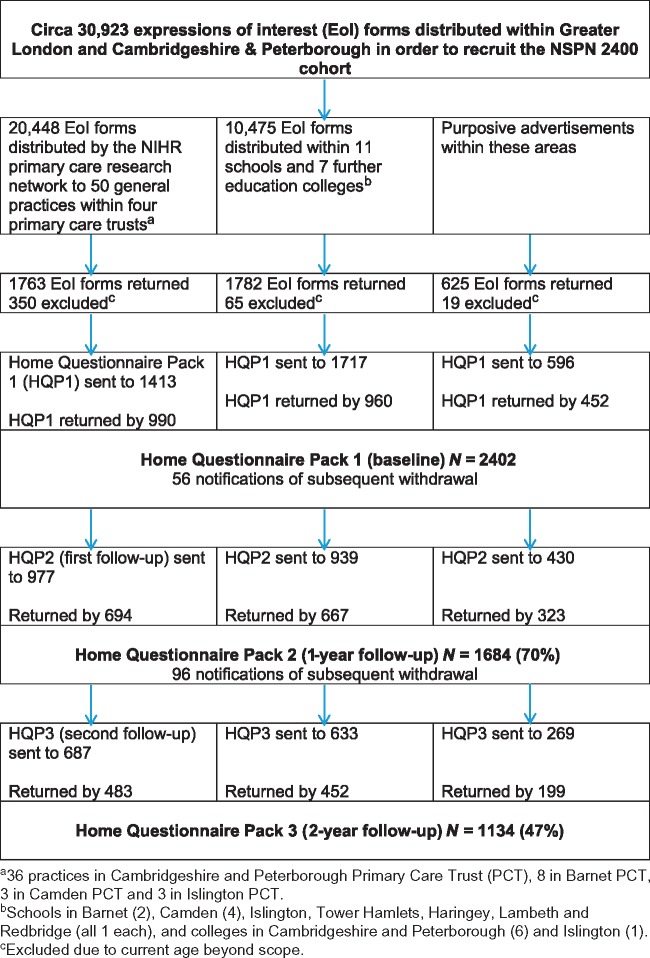
STROBE diagram showing the recruitment stages of the NSPN 2400 cohort. EoI = expression of interest; HQP = home questionnaire pack.

The STROBE diagram (Figure 2) shows that an estimated 30,923 EoI forms were distributed within GP’s practices and schools, of which 4170 (13.5%) were returned to the NSPN recruitment team. From the 4170 pool, 3726 people were eligible for further participation. 444 participants were rejected on the basis of the age- sex- strata being sufficiently populated. The Home Questionnaire Pack was sent to all eligible 3726 participants and returned by 65% of them (N = 2402, marking the baseline assessment stage of the NSPN 2400 Cohort.

## How often have they been followed up?

The NSPN 2400 Cohort is predicated on an accelerated longitudinal design (Figure 2). Thus, each participant has been invited to provide data on at least two occasions (at baseline and follow-up 1) through the completion of HQPs, and, ideally, on two occasions, thereafter; median interval for return of subsequent questionnaires (inter-quartile range) between baseline and first follow-up was 12 months (11–14 months), and between second and third assessments was 13 months (12-16 months). Figure 2 shows that follow-up 1 yielded a 70% response and follow-up 2 a 47% response rate when compared with HQP baseline. In total, HQP data at three time points were obtained from 1134 participants (as of December 2016). The reasons for non-response could not be determined as non-response equated to participants not returning the HQP; a minority of participants told us they did not want to take part further.

Each HQP follow-up was separated by an interval, described as the difference in days between the return date of HQP baseline and the return date of subsequent follow-up questionnaires. A median interval for HQP follow-up 1 was exactly 1 year (365 days) and the Inter-Quartile Range (IQR) was 85 days. For HQP follow-up 2 the median was 2.25 years (823 days) and the IQR was 120 days. The median interval between HQP follow-up 1 and 2 was 1.1 years (405 days) and the IQR was 114.5 days. Sociodemographic characteristics of those that dropped out at each follow-up are provided in Supplementary Materials section 1. In general, no obvious biases were observed in regards to ethnicity, place of birth, parental qualification and number of males/females for participants that did not complete follow-up questionnaires. Table 1 presents number of participants at follow-up 1 for the Cognition and MRI cohorts and time lag between assessments.

**Table 1:** Calculation of participants falling within each quantile (Q) based on the number of days it took them to return the HQP at each wave of assessment

**HQP baseline**	**Q1 (0-10 days)**	**Q2 (11-15 days)**	**Q3 (16-24 days)**	**Q4 (25-352 days)**
Total *N* = 2402	*N* = 601	*N* = 647	*N* = 565	*N* = 590
**HQP follow-up 1**	**Q1 (0-13 days)**	**Q2 (14-21 days)**	**Q3 (22-36 days)**	**Q4 (37-793 days)**
Total *N* = 1684	*N* = 464	*N* = 398	*N* = 403	*N* = 419
**HQP follow-up 2**	**Q1 (0-13 days)**	**Q2 (14-23 days)**	**Q3 (24-35 days)**	**Q4 (36-315 days)**
Total *N* = 1134	*N* = 293	*N* = 271	*N* = 283	*N* = 287

Median number of days from the date the questionnaire was sent to the date it was returned were calculated for each HQP wave. For HQP baseline the median was 15 days and the IQR was 14 days. For HQP follow-up 1 the median was 21 days and the IQR was 23 days. Finally, for the HQP follow-up 2 the median was 23 days and the IQR was 22 days. Table 2 presents number of the NSPN 2400 Cohort participants falling within each quantile using the 0.25%, 0.50% and 0.75% cut offs.

**Table 2:** Participant number and time-lag calculation between baseline and follow-up 1 for the cognition and MRI cohorts

	IUA baseline	IUA follow-up 1
Cognition cohort	*N* = 785	*N* = 568
		median time lag: 18.0 months, range: 11.8-31.4 months
MRI cohort	*N* = 318	*N* = 234
		median time lag: 15.4 months, range: 11.7-28.0 months

Cognition cohort retention was 72% and MRI cohort retention was 74%.

IUA, in-unit assessment.

A Microsoft Access-based Cohort Management System (CMS) was devised to store identifiable data (held on secure, password protected University of Cambridge servers in accordance with the Data Protection Act (1998)). Upon completion of relevant assessments, data for each participant was recorded/transferred to a database using the Research Electronic Data Capture (REDCap) software.^5^ Following successful transfer and quality checks, data were released for manipulation and analysis in an anonymised form to any researcher that was approved by Principal Investigators.

## What has been measured?

Table 3 below lists the self-report instruments included in Home Questionnaire Pack (HQP) to measure common mental health constructs by focusing on mood, behaviour and general well-being. The Sociodemographic Questionnaire (SQ) was primarily built to reflect questions asked within the 2011 public census to define participant’s family characteristics like ethnicity, highest maternal and/or paternal qualification, current postcode, employment status etc. If a participant was under the age of 18, parental consent was sought for them to participate in the study and complete the HQP. The SQ was completed by the parent if the participant was under-age.

**Table 3:** List of measures available in each NSPN cohort

NSPN 2400 cohort		HQP	HQP	HQP
		baseline	follow-up 1	follow-up 2
Moods and Feelings Questionnaire^1^		X	X	X
Revised Children’s Manifest Anxiety Scale+		X	X	X
Leyton Obsessional Inventory^3^		X	X	X
The Antisocial Behaviours Checklist^a^		X	X	X
Rosenberg Self-Esteem Scale^4^		X	X	X
Life Events Questionnaire^5^		X	X	X
Kessler Psychological Distress Scale^6^		X	X	X
Antisocial Process Screening Device^7^		X	X	X
Child and Adolescent Disposition Scale^8^		X	X	X
Drugs Alcohol and Self Injury^a^		X	X	X
Schizotypal Personality Questionnaire^9^		X	X	X
Warwick Edinburgh Mental Well-being Scale^10^		X	X	X
Inventory of Callous-Unemotional Traits^11^		X	X	X
Barratt Impulsive Scale^12^		X	X	X
Family Assessment Device (General Family Functioning subscale)^13^		X	X	X
Friendship Questionnaire^14^^,a^		X	X	X
Alabama Parenting Questionnaire^15^		X	X	–
Measure of Parenting Style^16^		X	X	–
Positive Parenting Questionnaire^a^		X	X	–
Affective Personalities Questionnaire ^7^^,^^18^^,a^		–	–	X
Reflective Function Questionnaire^19^		–	–	X
Sociodemographic Questionnaire^a^		X	X^b^	X^b^
Padual Inventory – Washington State University Revision^20^		–	X	X

**Cognition cohort**			**IUA baseline**	**IUA follow-up 1**

**Cognitive battery module**				
Orthogonalized Go-NoGo task^21^			X	X
Roulette task^22^			X	X
Human Approach-Avoidance task^23^			X	X
Information Gathering task^24^			X	X
Two-step task^25^			X	X
Delegated Intertemporal Discounting task^26^			X	X
Investor-Trustee task^27^^,^^28^			X	X
Subjective Well-being task^29^			X	X
**Clinical assessment module**				
Edinburgh Handedness Inventory^30^			X	–
Child Trauma Questionnaire^31^			X	X
Tanner Puberty Scale^32^			X	X
Hormone Question Sheet^a^			X	X
Wechsler Abbreviated Scale of Intelligence (WASI)^33^			X	X
Height, weight, waist circumference^a^			X	X
Self-report of youth behaviour^34^			–	X
Snaith Hamilton Pleasure Scale^35^			–	X
Obsessive Compulsive Inventory Revised^36^			–	X
SCID 1 (Depression, Suicidal, Mania, Substance Use)^37^			X	X
SCID 2 (PLIKS: Unusual experience, Hallucination)^37^			X	X
SCID 3 (PLIKS: Delusions)^37^			X	X
SCID 4 (Others)^37^			X	X

**Figure 3:**
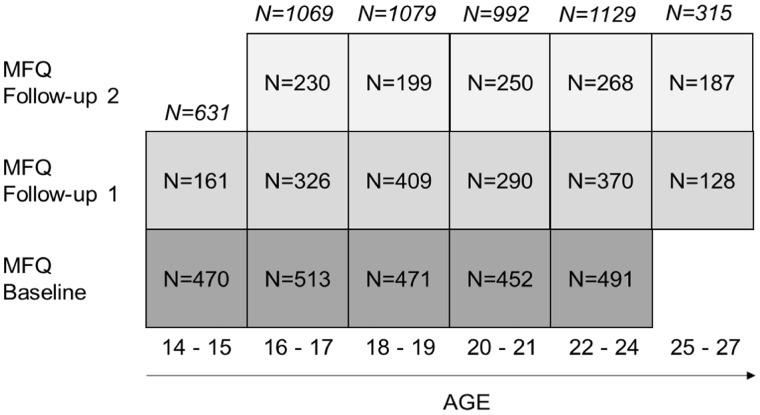
Illustration of number of participants who completed Moods and Feeling Questionnaire (MFQ) within each age group at each stage of recruitment (as of October 2016). Italicised N indicate the total number of participants for each age group. Abbreviations: MFQ = Moods and Feelings Questionnaire.

Measures for the MRI and cognition cohorts are split in Table 3 to reflect the modular approach to in-unit assessments. Detailed description of both cognitive task battery and MRI acquisitions are provided in Supplementary Materials section 2. Figure 3 is an example of number of participants for each age bin that completed Moods and Feelings Questionnaire (MFQ) as part of HQP.

## What has it found? Key findings and publications

### The NSPN 2400 Cohort representativeness

To assess the representativeness of the NSPN 2400 Cohort in terms of the England & Wales youth population, five sociodemographic characteristics were compared with data from the 2011 census extracted from the labour market tables produced by the Office of National Statistics (data queries were run on www.nomisweb.co.uk). Detailed explanation and figures can be found in Supplementary Materials section 3. In summary, the NSPN Cohort: 1) broadly matched the ethnicity to the general population of England & Wales, with mixed and Asian groups slightly over-represented; 2) closely resembled the England & Wales population structure when looking at the proportion of UK vs. non-UK births; 3) NSPN volunteers’ parents were more likely to complete qualifications, which translates to an almost 10% difference in achieving Level 1 to 4 qualification when compared with England & Wales. The percentage of vocational qualifications achieved was very similar; 4) on average across the ages there were 5% more females and 5% fewer males compared to England & Wales; 5) an under-representation within the lowest 1^st^ decile and an over-representation within the 9^th^ highest decile was observed when compared to the distribution of Indicator of Multiple Deprivation^6^ ranks in England. The remaining deciles are broadly comparable to England.

### Adolescence is associated with genomically patterned consolidation of the hubs of the human brain connectome

As an example of the kind of work linking the cohort with the biological measures in the sub-groups, we have studied developmental changes in the cerebral cortex. We found, consistently in two MRI cohorts, that human brain changes in adolescence were concentrated on the more densely connected hubs of the connectome. These particularly well connected regions were located in association cortex, parts of the brain that support higher order cognitive and social processing. At age 14, hub regions had lower magnetisation transfer (MT) than other cortical areas, indicating lower myelin content, but had greater increases in this measure during the 14 to 24 year period. This suggests that cortical hubs have more prolonged myelination that the rest of the cortex. This topologically focused process of cortical consolidation was associated with expression of genes enriched for normal synaptic and myelin-related processes and risk of schizophrenia. We conclude that consolidation of anatomical network hubs could be important for normal and potentially different for clinically disordered adolescent brain development.^7^

### Gene transcription profiles associated with inter-modular hubs and connection distance in human functional magnetic resonance imaging networks

Human functional magnetic resonance imaging (fMRI) brain networks have a complex topology comprising integrative components, e.g. long-distance inter-modular edges that are theoretically associated with higher biological cost. We estimated intra-modular degree, inter-modular degree and connection distance for each of 285 cortical nodes in multi-echo fMRI data from 38 healthy adults and matched our neuroimaging data with openly available transcriptomic expression measures of more than 20,000 genes. We showed nodes in superior and lateral cortex with high inter-modular degree and long connection distance had local transcriptional profiles enriched for oxidative metabolism and mitochondria, and for genes specific to supragranular layers of human cortex. In contrast, primary and secondary sensory cortical nodes in posterior cortex with high intra-modular degree and short connection distance had transcriptional profiles enriched for RNA translation and nuclear components. We conclude that topologically integrative hubs, mediating long-distance connections between modules, are more costly in terms of mitochondrial glucose metabolism.^8^

### Impulsivity and peer influence study

This was the first study analysing data from the cognition cohort. We found that inter-temporal discounting,^9^ a standard measure of impulsivity in animal and human research, was subject to peer influence even if social or monetary rewards did not motivate participants. Participants shifted their level of impulsivity towards that of experimental ‘partners’ depending on two key characteristics: first, how relevant they felt their partner’s observed choices were; and second, how certain they were about their own tastes in the matter.^10^

## What are the main strengths and weaknesses?

### Strengths

To our knowledge, the NSPN 2400 Cohort is the first to combine the behavioural, cognitive and neuroimaging measures to study the normative development of well-being and mental health in an adolescent/young adult cohort representing the England and Wales general population. Despite the NSPN 2400 being a volunteer sample, we demonstrated that it is broadly representative of the England & Wales youth; therefore, it is reasonable to generalise research findings to a wider population. The accelerated longitudinal design will allow estimation of development (growth curves) describing how self-report, cognitive or MRI measures change as a function of chronological age and gender, and to sketch the developmental trajectory of mental health. To do this, mixed effects models will be used to analyse outcome data, using fixed and random effects for linear and quadratic terms for age, with stratification by gender given that differences between boys and girls are accepted within the relevant literature.

Another strength is a relatively good retention rate in the study, particularly at the first follow-up. Currently reported 47% retention rate for the second follow-up may increase as data collection continues.

### Weaknesses

A paradoxical weakness is that participants were volunteers for an intensive study, albeit drawn from a randomly selected population, and volunteers are a unique population, especially psychologically. This sampling bias is perhaps evident as participants were from families with higher parental educational attainment when compared with the general population. This potentially means that, for younger participants in particular, they were encouraged to take part by parents particularly aware of the importance of research. That said, many participants are older and more autonomous. Unfortunately, we did not seek ethical committee approval to collect information on people who expressed interest in the study but did not, subsequently, consent to take part. Furthermore, we were not able to obtain accurate estimations of the population-based sampling frame (e.g. numbers of people in age-sex GP registers) from the PCRN, and we attempted to follow at two years only those we had measured at the one-year follow-up, standard in an accelerated design. Another limitation is that we have no information on the important period of change before the age of 14 years; this intend this to be the focus of further work. Despite best efforts, 53% attrition also means that we do not have the longitudinal information on every participant, which decreases our power to detect long-terms effects and introduces bias.

Finally, the cohort is, by design, yet to live through the main period of risk for incident mental illness. Thus, the current emphasis is on characterising developmental styles and variations in the quantitative behavioural, cognitive and neural domains included in the study. It will be some time before the participants are at an age when the full implications of these differences will be known in terms of risk of conventional diagnostic categories. However, the intention is to describe and model developmental processes that transcend these unsatisfactory concepts.

### Can I get hold of the data? Where can I find out more?

The study is committed to open science with the aim to make the anonymised dataset fully available to the research community. The participants have consented to their de-identified data being made available to other researchers. The first step has been to define a concise application process that establishes the *bone fides* of those making the request, accessible by email to openNSPN@medschl.cam.ac.uk. Requests are reviewed by the investigators. Second, data sets used for all publications involving NSPN are available at URLs to be included in the publication. Finally, the study aspires to making data publically available. This publication is based on data at https://doi.org/10.17863/CAM.12547. A process has begun involving participants themselves, ethicists, the funder, lawyers and experts in informatics and research governance in order to establish a framework in which to move as far as possible towards that aspiration.

Profile in a nutshell

- The NSPN 2400 Cohort was established to link normal and psychopathological variation at the behavioural, cognitive and emotion level to phenotypic variation at the level of brain systems, subverting the unhelpful division between adult and child/adolescent psychiatry by measuring specified dimensions in healthy volunteers in the age range of 14–24 years.
- Participants were recruited in 2012 from Greater London and Cambridgeshire and are broadly representative of England & Wales.
- Self-reported behavioural data are available at three time-points with questionnaire return rate of 70% at one year follow-up, and 47% at two years when compared with baseline participant number of N = 2402.
- Cognitive battery data retention rate is 72% and for MRI data is 74% at follow-up 1, with baseline data points for 785 and 318 participants respectively.
- The NIHR Cambridge BioResource extracted and stores DNA from 2087 saliva samples. Part of each sample will be genotyped using the UK Biobank Axiom Array. This comprises 820,967 genetic markers designed for three domains: markers of specific interest, rare coding variants, and genome-wide coverage.
- The NSPN 2400 Cohort (measures of mental well-being, demographics and DNA), Cognitive cohort (cognitive tasks measures and clinical assessment) and MRI cohort (structural and functional imaging measures) data will be accessible for collaboration upon agreement with the principal investigators. Enquiries should be submitted to openNSPN@medschl.cam.ac.uk.

## Funding

This study was supported by the Neuroscience in Psychiatry Network, a strategic award from the Wellcome Trust to the University of Cambridge and University College London (095844/Z/11/Z). Additional support was provided by the National Institute for Health (NIHR) Research Cambridge Biomedical Research Centre, the NIHR Collaboration for Leadership in Applied Health Research & Care East of England, and the Medical Research Council (MRC)/Wellcome Trust Behavioural and Clinical Neuroscience Institute.

## Supplementary Material

Supplementary Data
