# Supplementary material for: Cohort Profile: The NSPN 2400 Cohort: a developmental sample supporting the Wellcome Trust NeuroScience in Psychiatry Network
Source: Int J Epidemiol. 2017 Nov 21;47(1):18–19g. doi: 10.1093/ije/dyx117 (PMC5837633; doi:10.1093/ije/dyx117)
Supplement: Supplementary Data [file dyx117_ije-2016-12-1474-file004.docx]

Supplementary Materials:

Section 1

HQP follow-up 1 questionnaire was not returned by 718 participants (3 participants were missing information on ethnicity, 6 on country of birth, and 93 on parental highest qualification).

HQP follow-up 2 questionnaire was not returned by 550 participants (1 participant was missing information on ethnicity, 1 on country of birth, 58 on parental highest qualification).

**Figure 4** Comparison of ethnical composition of participants that have dropped out from the NSPN 2400 Cohort at follow-up 1 (N=718) and follow-up 2 (N=550) compared with ethnic composition at HQP baseline (N=2402).

**Figure 5** Comparison of proportion of participants classified as UK vs. non-UK birth at HQP baseline and follow-up 1 and 2.

**Figure 6** Comparison of parental highest qualification characteristic for participants across HQP baseline, those lost at HQP follow-up 1 and 2.

| **Lost at follow-up 1** | 14-15 | 16-17 | 18-19 | 20-21 | 22-24 | overall |
| --- | --- | --- | --- | --- | --- | --- |
| Total | 111 (15%) | 158 (22%) | 159 (22%) | 148 (21%) | 143 (20%) | - |
| Females | 58 (53%) | 71 (45%) | 75 (47%) | 64 (43%) | 54 (38%) | 45% |
| Males | 52 (47%) | 87 (55%) | 84 (53%) | 84 (57%) | 89 (62%) | 55% |
| **Lost at follow-up 2** | 14-15 | 16-17 | 18-19 | 20-21 | 22-24 | overall |
| Total | 113 (20%) | 149 (26%) | 100 (17%) | 107 (18%) | 110 (19%) | - |
| Females | 60 (53%) | 72 (48%) | 46 (46%) | 58 (54%) | 53 (48%) | 50% |
| Males | 53 (47%) | 77 (52%) | 54 (54%) | 49 (46%) | 57 (52%) | 50% |

**Table 4** Comparison of the proportion of males and females lost at follow-up 1 and 2 per age stratum.

| Section 2 | | |
| --- | --- | --- |
| Task (with key reference) | Brief description of key constructs assessed | Key derived measures. |
| **Orthogonalized Go-NoGo task** (1) | Pavlovian biases to engage in action in order to obtain rewards and to abstain from action to avoid losses; Motivational power of rewards vs. Losses; Learning rate of the habitual system. | **Pavlovian Bias**;  **Sensitivity to appetitive outcomes** and sensitivity to aversive outcomes (or equivalently, inverse-decision-temperatures to the same).  General bias for action rather than non-action;  Motivation-independent, 'irreducible', variability in decision-making;  and learning rate. |
| **Roulette task, assessing Economic Risk-Preference** (2) | Risk-avoidance (preference for outcome distributions of low variance). | **Baseline preference for taking gambles;**  Preference weight for variance, compared to the mean, of an outcome distribution, named '**Economic risk preferenc**e';  Effect of the asymmetry of outcome distribution on preferences. |
| **Approach-Avoidance conflict task** (3) | Willingness to expose oneself to different levels of risk for the sake of amassing rewards. | Risk-taking levels overall, named '**Overt Risk Taking**';  How risk-taking is modulated by increasing hazards within each context, named '**Within context risk modulation**' |
| **Two-Step task** (4) | Strength of habitual ('model-free') and goal-directed ('model-based') decision-making | **Goal-directedness**: shift in decisions as a consequence of a different decision being more advantageous according to the transition probabilities inherent in the task. **Habit-basedness**: shift in decisions as a consequence of the last action being rewarded. |
| **Information Gathering task** (5) | Assessment of whether future decisions will on balance be more advantageous if one gathers more information. | **Information Sampling noise**, which determines not only decision variability but also effective depth of planning.  Subjective cost of every piece of information asked for. |
| **Delegated Intertemporal-Discounting task** (6) | Baseline inter-temporal discounting; shift in discounting preferences upon exposure to peers' preferences. | **Basic hyperbolic temporal discounting** coefficient;  Relevance of others' observed preferences to the self;  **Discounting taste uncertainty**, i.e. uncertainty about one's own tastes in this domain. |
| **Investor-Trustee task**  (7,8) | Overall strategies used to elicit cooperation and avoid being exploited by the Investor in this task. | **Initial trust**, i.e. the amount given by the investor to the Trustee before they have any specific information about them.  **Trust-building**: A more complex measure pertaining to the style of exchange established. This is a descriptive measure of the degree to which Investor and Trustee tended to respond to reductions (or increases) in each other's contributions by retaliating (or exploiting, etc.). |
| **Subjective Wellbeing Task** (9) | How profit and loss arising from a decision with uncertain outcomes influences momentary self-reported wellbeing (‘momentary happiness’) | **Baseline momentary happiness**  Effect of expectations on momentary happiness.  Effect of expectation violations (prediction errors) on momentary happiness. |

**Table 5** Cognitive task battery description. Key measures derived from computational models are shown in bold, but this is not a final list. Derived measures are still being refined.

| MRI acquisition (with key reference) | Brief description of acquisition | Key derived measures |
| --- | --- | --- |
| **Multi-echo echo-planar imaging** (10) | N=270 whole brain echo-planar imaging acquisitions at three echo times (13, 30.55 and 48.1ms) at 3.75mm^3^ isotropic resolution. Repetition time of 2.42s leading to a total scan time of 11 minutes. | Voxel-based 4 dimensional maps (brain volumes at multiple times points) of blood oxygen level dependent (BOLD) signal intensity after processing to remove artefactual and non-biological variance. |
| **Multi-parametric mapping** (11) | Longitudinal relaxation time weighted (T1w) image, magnetistation transfer weighted (MTw) image, and a proton density (PDw) weighted image, and two field maps. All acquisitions collected at 1mm^3^ isotropic resolution. Total scan time 24 minutes. | Voxel-based 3 dimensional quantitative maps of longitudinal relaxation rate (R1), effective transverse relaxation rate (R2*), magnetisation transfer (MT) and effective proton density (PD*). |
| **Diffusion weighted imaging** (12) | N=63 diffusion weighted images collected at 2mm^3^ isotropic resolution. Total scan time 11 minutes. | Voxel-based 3 dimensional maps of fractional anisotropy, mean diffusivity, axial and radial diffusivity. |

**Table 6** Magnetic Resonance Imaging detailed description of acquisitions.

Section 3

*Ethnicity*

The Sociodemographic Questionnaire was designed to identify the same five self-ascribed ethnic subgroups as the 2011 census: White (including English/Welsh/Scottish/Northern Irish/British, Irish, Gypsy or Irish Traveller, any other white background; Mixed (White and Black Caribbean, White and Black African, White and Asian, any other multiple ethnic background); Asian (Indian, Pakistani, Bangladeshi, Chinese, any other Asian background); Black (including African, Caribbean, any other black background); Other (Arab, any other ethnic group). Figure 3 below illustrates proportion of England & Wales citizens age 14 to 24.99 that fall within each ethnic category in comparison to the NSPN 2400 Cohort (N=2402, ethnicity missing data N=3). It can be observed that the NSPN 2400 Cohort broadly matched the ethnicity to the general population of England & Wales with mixed and Asian groups slightly over-represented.

**Figure 7** Ethnic composition.

*Country of birth*

Each NSPN 2400 Cohort participant was asked to indicate where she/he was born; responses are compared with the proportion of UK vs. non-UK births within England & Wales in Figure 4. Of the 2402 baseline data, 7 participants did not answer question about country of birth. Just as it was the case with ethnicity, the NSPN 2400 Cohort closely resembles the England & Wales population structure.

**Figure 8** UK vs. non-UK birth composition.

*Parental education*

In 2011 census parental education level is reported as the highest level of qualification achieved. Considering the age of the NSPN 2400 Cohort participants (14-24), census parental education data were extracted for ages 34 to 64 under assumption that the youngest parent would be around 20 and the oldest 40 years old when the participant was born. We collapse three levels (Levels 1-3) into one group due to lack of granular data on specific examination grades parents achieved at GCSE or A Level, which is the driving detail needed for specific level assignment. Thus, the group “Level 1-3” includes a parent whose highest qualification was at minimum a GCSE and/or an A level qualification at any grade. Level 4 group included parents with at least a first degree (or equivalent) and, at most, a doctoral degree. There were 182 participants with missing data for parental education within the NSPN 2400 Cohort.

It can be concluded that parents of the NSPN 2400 Cohort volunteers were more likely to complete educational qualifications, which translates to an almost 10% difference in achieving Level 1 to 4 qualification when compared with England & Wales. However, the percentage of vocational qualifications achieved was very similar.

**Figure 9** Distribution of parental education.

*Proportion of males and females in each age group*

Figure 6 demonstrates that across the 5 age groups to which females and males were recruited, there is a systematic increased voluntary participation of females. When compared to England & Wales there were on average 5% more females than in the general population, which is characterised by an almost equal gender split. There were on average 5% fewer males in the NSPN 2400 Cohort compared to England & Wales population and this difference rose to 8% when compared to the proportion of females recruited to the NSPN 2400 Cohort.

**Figure 10** Age-specific proportion of females and males.

*Indicator of Multiple Deprivation (IMD)*

IMD is the official measure of relative deprivation for small areas in England (of around 1500 citizens, defined as a Lower-layer Super Output Areas [LSOA]) and gives an indicative measure of individual deprivation based on postcode of participants’ addresses. There are 32842 LSOA’s in England in 2010, the higher the rank the lower the deprivation of a given area. It is common to describe the level of deprivation by estimating whether an LSOA falls among the most deprived 10%, 20% or 30% areas in England. However, there is no definitive cut-off at which an area is described as ‘deprived’. In the NSPN 2400 Cohort, postcode at entry to the study was used to extract the relative IMD rank for each participant. The split of participants that belong to a specific deprivation percentile are shown in Table 3. Five NSPN 2400 Cohort participants were missing IMD rank. It can be noted that in general there is an under-representation within the lowest 1^st^ decile and an over-representation within the 9^th^ highest decile within the NSPN 2400 Cohort when compared to the distribution of IMD ranks in England. The remaining deciles are broadly comparable to IMD ranks within England.

| IMD rank | 10% | 20% | 30% | 40% | 50% | 60% | 70% | 80% | 90% | 100% |
| --- | --- | --- | --- | --- | --- | --- | --- | --- | --- | --- |
| NSPN 2400 LSOA N=2399 | 129  5% | 221  9% | 159  7% | 183  8% | 221  9% | 237  11% | 223  9% | 312  13% | 464  19% | 248  10% |
| England LSOA N=32482 | 3249  10% | 3248  10% | 3248  10% | 3248  10% | 3248  10% | 3248  10% | 3248  10% | 3248  10% | 3248  10% | 3249  10% |

**Table 7** Socioeconomic representativeness of NSPN 2400 Cohort compared with England: number of participants falling within each decile group for Index of Multiple Deprivation (IMD) rank.

References:

1. Guitart-Masip M, Huys QJM, Fuentemilla L, Dayan P, Duzel E, Dolan RJ. Go and no-go learning in reward and punishment: Interactions between affect and effect. Neuroimage 2012;62(1):154–66.

2. Symmonds M, Wright ND, Bach DR, Dolan RJ. Deconstructing risk: Separable encoding of variance and skewness in the brain. Neuroimage 2011;58(4):1139–49.

3. Bach DR, Guitart-Masip M, Packard PA, Miró J, Falip M, Fuentemilla L, et al. Human hippocampus arbitrates approach-avoidance conflict. Curr Biol 2014;24(5):541–7.

4. Daw ND, Gershman SJ, Seymour B, Dayan P, Dolan RJ. Model-based influences on humans’ choices and striatal prediction errors. Neuron 2011;69(6):1204–15.

5. Moutoussis M, Bentall RP, El-Deredy W, Dayan P. Bayesian modelling of Jumping-to-Conclusions bias in delusional patients. Cogn Neuropsychiatry 2011;16(5):422–47.

6. Moutoussis M, Dolan RJ, Dayan P. How People Use Social Information to Find out What to Want in the Paradigmatic Case of Inter-temporal Preferences. PLoS Comput Biol 2016;12(7):e1004965.

7. Fett AKJ, Shergill SS, Joyce DW, Riedl A, Strobel M, Gromann PM, et al. To trust or not to trust: The dynamics of social interaction in psychosis. Brain 2012;135(3):976–84.

8. King-Casas B, Sharp C, Lomax-Bream L, Lohrenz T, Fonagy P, Montague PR. The rupture and repair of cooperation in borderline personality disorder. Science 2008;321(5890):806–10.

9. Rutledge RB, Skandali N, Dayan P, Dolan RJ. A computational and neural model of momentary subjective well-being. Proc Natl Acad Sci 2014;111(33):12252-12257.

10. Kundu P, Inati SJ, Evans JW, Luh W-M, Bandettini PA. Differentiating {BOLD} and non-BOLD signals in fMRI time series using multi-echo {EPI}. Neuroimage 2012;60(3):1759–70.

11. Weiskopf N, Suckling J, Williams G, Correia M. MM, Inkster B, Tait R, et al. Quantitative multi-parameter mapping of R1, PD*, MT, and R2* at 3T: A multi-center validation. Front Neurosci 2013;7:95. doi: 10.3389/fnins.2013.00095.

12. Beaulieu C. The basis of anisotropic water diffusion in the nervous system - A technical review. NMR in Biomedicine 2002; p. 435–55.
